# Expression of metastasis suppressor 1 in cervical carcinoma and the clinical significance

**DOI:** 10.3892/ol.2014.2508

**Published:** 2014-09-08

**Authors:** JUAN ZHANG, YING TONG, LI REN, CHUN-DONG LI

**Affiliations:** 1 Department of Obstetrics and Gynecology, Airforce General Hospital, Beijing 100142, P.R. China; 2 Department of Pathology, Airforce General Hospital, Beijing 100142, P.R. China

**Keywords:** cervical malignancy, cervical intraepithelial neoplasia, metastasis suppressor 1

## Abstract

This study aimed to investigate the expression of metastasis suppressor 1 (MTSS1) in cervical intraepithelial neoplasia (CIN) and malignant cervical tissues, and the role of MTSS1 in carcinogenesis. MTSS1 expression was detected by immunohistochemistry in 147 cervical tissue specimens collected from 30 healthy individuals, 30 patients with cervical CIN I, 30 patients with CIN II–III and 57 patients with cervical cancer. The association between MTSS1 expression and clinicopathological factors was also examined. MTSS1 was found to be positively expressed in 43.33% CIN I cervical tissues, 100% CIN II–III cervical tissues and 100% malignant cervical tissues, but was weakly or negatively expressed in benign cervical tissues. The positive expression rates of MTSS1 were significantly higher in CIN II–III and malignant cervical tissues than in CIN I or normal cervical tissues (P<0.05). When examining MTSS1 expression and clinicopathological factors, the strong positive MTSS1 expression rates in early-stage versus middle- and advanced-stage cervical cancer tissues were 39.13% and 82.35%, respectively. Furthermore, the positive expression rates of MTSS1 were significantly higher in cervical tissues at an advanced clinical stage than those at an early clinical stage (P<0.05). The results suggest that the dysregulation of MTSS1 may be involved in cervical carcinogenesis, and thus MTSS1 may be a novel diagnostic biomarker or therapeutic target in cervical cancer patients.

## Introduction

Study has confirmed that persistent infection with high-risk type human papilloma virus (HPV) may result in the gradual alteration of normal cervical epithelial tissue to cervical intraepithelial neoplasia (CIN), which may progress to cervical invasive carcinoma (1). However, the underlying pathogenetic mechanism of cervical cancer, which may be a multigene, multifactor, multistep and multistage complex process, remains unclear. Previous studies have demonstrated that the coordinated regulation of cytoskeletal proteins is pivotal in motility, invasion and metastasis (2–4). Metastasis suppressor 1 (MTSS1), also termed missing in metastasis (MIM), is a newly identified actin binding protein that is mainly involved in cytoskeletal remodeling, signal transduction and transcriptional activation, and is closely associated with tumor growth and invasion (5). However, the association between MTSS1 and cervical lesions has not yet been reported. In the present study, the role of MTSS1 in cervical carcinogenesis was examined by investigating MTSS1 expression in pre-cancerous cervical lesions and malignant cervical tissues, and by analyzing the association between MTSS1 expression and clinicopathological factors.

## Materials and methods

### Clinical materials

The study included a total of 60 patients with CIN (30 with CIN I and 30 with CIN II–III) and 57 patients with cervical cancer, as well as 30 healthy individuals. The mean (± SD) age of all participants was 44.36±10.53 years. Cervical pathological biopsy specimens were surgically removed during cervical biopsy and radical hysterectomy performed between January 2000 and December 2012 in outpatient services at the Airforce General Hospital (Beijing, China). All specimens were maintained in tissue paraffin blocks. The pathological diagnosis was reviewed in a double-blind manner by two experienced pathologists. The cervical cancer clinical stage was defined according to the criteria determined by the International Federation of Gynecology and Obstetrics in 2009 (6): 23 cases were early-stage cervical cancer (stage I–IIa) and 34 cases were advanced-stage (stage IIb–IV). With regard to the degree of differentiation, 12 cases were well-differentiated, 35 cases were moderately differentiated and 10 cases were poorly differentiated. Lymph node metastasis status was examined, and 48 cases without lymph node metastasis and 9 cases with lymph node metastasis were detected. A total of 57 patients were diagnosed with cervical squamous carcinoma and 30 cases of normal cervical epithelium were selected for comparison. No significant differences between groups with regard to general patient information, including age and pregnancy history, were identified. This study was approved by the ethics committee of Airforce General Hospital (Beijing, China). Written informed consent was obtained from all patients.

### Immunohistochemical staining of MTSS1 protein

MTSS1 expression in the 147 cervical tissue specimens was detected by immunohistochemistry using the EnVision two-step immunohistochemical staining method (Dako, Carpinteria, CA, USA). The anti-MTSS1 primary antibody used was ab56780 (1:130 dilution; Abcam, Cambridge, UK). An EnVision immunohistochemical kit (secondary antibody, K5007, DAB chromogenic system) was purchased from Beijing Golden Bridge Biotechnology Company (Beijing, China). Normal cervical tissues were selected to serve as a positive control. Phosphate-buffered saline (PBS; Beijing Zhongshan Golden Bridge Biotechnology Co., Ltd., Beijing, China) served as a negative control in place of the primary antibody. For each case, 3-μm sections were cut from the paraffin-embedded tumor tissue blocks and placed on slides. The tissues were de-waxed in xylene and rehydrated in alcohol. Antigen retrieval was performed by placing the tissues in sodium citrate buffer (Beijing Zhongshan Golden Bridge Biotechnology Co., Ltd.) and applying a high voltage for 3 min (pH 6.0), followed by natural cooling. The sections were then placed in 3% H_2_O_2_ (Beijing Zhongshan Golden Bridge Biotechnology Co., Ltd.) for 10 min to inhibit endogenous peroxide activity, washed with distilled water and washed with PBS for 3 min (four times). The samples were then incubated in MTSS1 antibody at 37°C for 45 min. Subsequently, the sections were washed with distilled water, then washed with PBS for 3 min (four times). The samples were then incubated with the secondary antibodies at 37°C for 25 min. Subsequently, the sections were washed with distilled water and PBS for 3 min (four times). Freshly prepared DAB chromogen was dropped onto the slides, which were then incubated at 37°C for between 3 and 5 min. The sections were then counterstained in hematoxylin (Beijing Yili Fine Chemicals, Co., Ltd., Beijing, China) (30 sec), washed with distilled water, differentiated with 1% hydrochloric acid alcohol, washed with distilled water and dehydrated in ascending grades of methanol (Beijing Yili Fine Chemicals, Co., Ltd.) prior to clearing in xylene (Beijing Yili Fine Chemicals, Co., Ltd.) and mounting under a cover slip.

### Immunohistochemical result determination

Positive reaction products were indicated by yellow, tan or brown particulates in the cytoplasm. Each section was analyzed by two pathologists who selected five fields under high-power magnification and scored each field according to the staining intensity and the percentage of positive cells (secondary scoring method). The number, expression intensity, shape and distribution characteristics of the brown particles were observed with an optical microscope [Nikon ECLIPSE 80i; Nikon Instruments (Shanghai) Co., Ltd., Shanghai, China]. The coloring intensity of cells was graded as 0 (no staining), 1 (faint yellow), 2 (yellow or deep yellow) or 3 (tan or brown). The specimens were also grouped into categories as determined by the percentage of positive cells: 0 = 0–4%, 1 = 5–25%, 2 = 26–50%, 3 = 51–75% or 4 = 76–100%. When the product of the staining intensity and the percentage of positive cells was >3, the specimen was defined as immune-positive. The staining results were divided into four grades according to the product score: − (negative, 0–2), + (weakly positive, 3–5), ++ (moderately positive, 6–8) or +++ (strong positive, 9–12).

### Statistical analysis

All statistical analyses were performed using SPSS 20.0 software (IBM, Armonk, NY, USA). Comparisons between groups were analyzed using χ^2^ tests and Fisher’s exact probability tests. P<0.05 was considered to indicate a statistically significant difference.

## Results

### MTSS1 expression in cervical tissues

**Figure 1:**
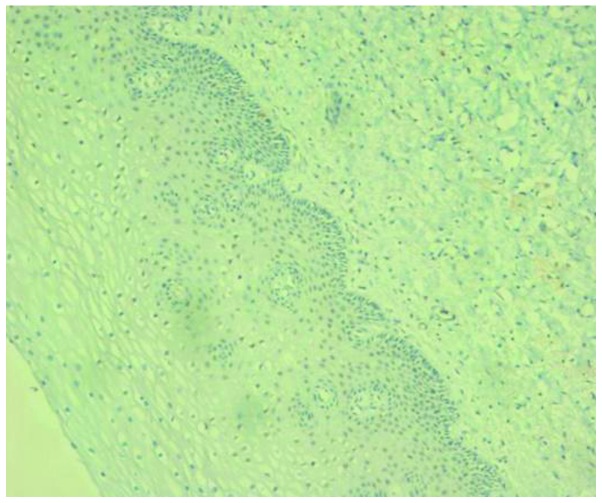
No expression of metastasis suppressor 1 was detected in normal cervical tissue.

**Figure 2:**
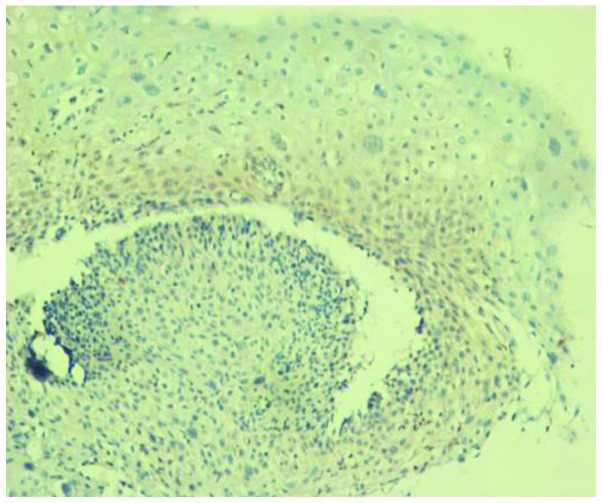
Weakly positive expression of metastasis suppressor 1 in cervical intraepithelial neoplasia I tissue.

**Figure 3:**
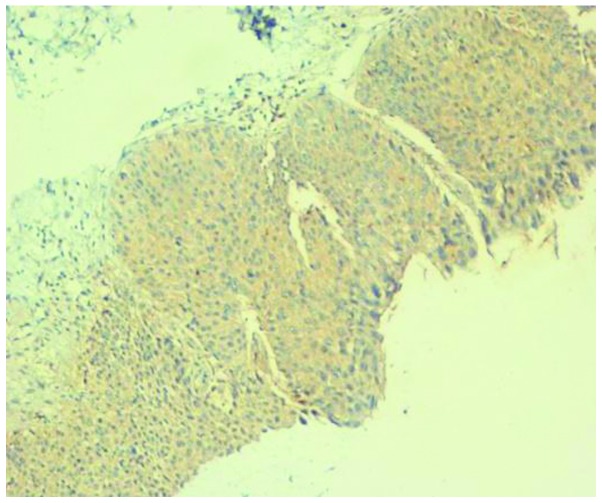
Moderate to strong positive expression of metastasis suppressor 1 in cervical intraepithelial neoplasia II–III tissue.

**Figure 4:**
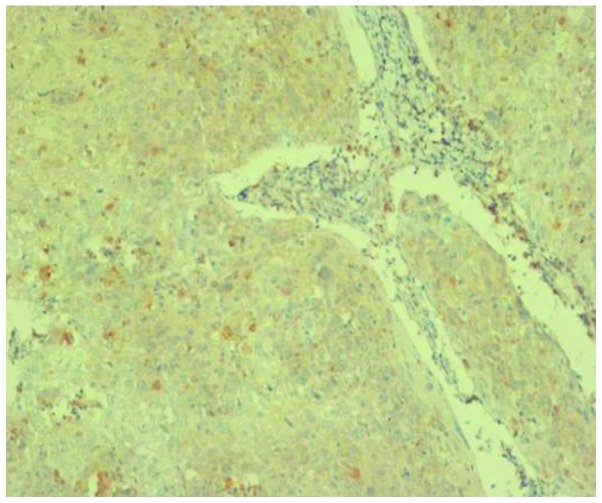
Moderate to strong positive expression of metastasis suppressor 1 in early-stage cervical carcinoma.

**Figure 5:**
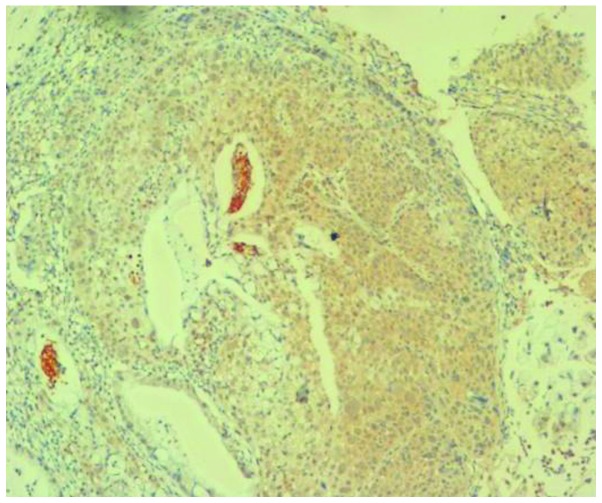
Strong positive expression of metastasis suppressor 1 in middle- and advanced-stage cervical carcinoma.

MTSS1 expression in the cytoplasm of benign cervical epithelial cells was predominantly negative or occasionally weakly positive. Approximately one-third of the cells in the CIN I specimens exhibited weakly positive staining for MTSS1 in the epithelium. The epithelial cells in the CIN II–III specimens exhibited moderate to strong MTSS1 expression, and expression was detected over the entire epithelial layer (Figs. 1–5). Within the cervical cancer group, early-stage cervical cancer tissues exhibited moderate to strong MTSS1 expression, and middle- and advanced-stage cervical cancer tissues exhibited strong positive MTSS1 expression.

Cytoplasmic expression of MTSS1 was significantly greater in cervical carcinoma and CIN II–III tissues than in normal cervical tissues (χ^2^=40.000, P<0.01). The MTSS1 positive expression rate in CIN II–III tissues was also significantly higher than that of the CIN I tissues (χ^2^=23.721, P<0.01). However, no significant differences in cytoplasmic MTSS1 expression were identified between CIN I and normal cervical tissues (χ^2^=3.774, P>0.05; Table I). However, cytoplasmic expression of MTSS1 was significantly higher in cervical carcinoma tissues when compared with normal cervical tissues (χ^2^=62.971, P<0.001).

### Correlation between MTSS1 expression and clinicopathological characteristics of cervical cancer

Among the 57 cases of cervical squamous carcinoma, the strong positive MTSS1 expression rate was significantly higher in middle- and advanced-stage cervical cancer specimens than in early stage cervical cancer specimens (χ^2^=42.043, P<0.05). However, the strong positive expression rate of MTSS1 was not correlated with patient age, tumor differentiation or the presence of lymphatic metastasis (P>0.05, Table II).

## Discussion

MTSS1, which maps to the 8q24.1 chromosomal region, is a gene that was first identified by Lee *et al* (7) when mRNA differential display technology was used to investigate the metastatic mechanism of bladder cancer. MTSS1 is expressed in normal bladder tissue and non-metastatic bladder cancer, but is deleted in metastatic bladder cancer and, therefore, was originally defined as a metastasis suppressor gene. Studies examining prostate cancer have also revealed that the overexpression of MTSS1 clearly inhibits cell metastasis, growth and adherence (8,9). Xie *et al* (10) demonstrated that MTSS1 expression levels in esophageal squamous cell carcinoma patients were significantly lower in high TNM stage tumors than in low TNM stage tumors, and MTSS1 expression levels were also lower in patients with lymph node metastasis than in those without. A gastric cancer study in a Chinese population also revealed that the expression levels of MTSS1 were increased in tissues adjacent to carcinoma, but were clearly reduced in cancer tissues (11). In addition, the MTSS1 expression levels were observed to be gradually reduced with decreasing degrees of histological differentiation. Decreased MTSS1 expression levels were significantly correlated with larger tumor size, deeper invasion, increased lymphatic metastasis and advanced tumor stage (11). In a study on human breast cancer, strong positive expression of MTSS1 was detected in normal breast tissue, which was significantly reduced or even completely absent in breast cancer tissues, and increased levels of MTSS1 expression were found to inhibit breast cancer cell growth, invasion and metastatic potential (12). These studies suggest that MTSS1 acts as a tumor metastasis suppressor gene in these malignancies.

In recent years, studies have found that MTSS1 exhibits tissue-specific expression, and increased MTSS1 expression has been associated with tumorigenesis in certain other types of malignant tumor, such as hepatocellular carcinoma (13), colorectal cancer (14), and head and neck squamous cell carcinoma (15). Ma *et al* (13) proposed that MIM-B (a MIM homologous isomer) may be an early predictor of liver cancer. Mattila *et al* (16) suggested that, instead of acting as a tumor metastasis suppressor gene, MTSS1 is a type of scaffolding protein that interacts with the oncogene Rac, actin and pseudopodia formation-related proteins. Lee *et al* (7) observed low expression levels of MTSS1 in the uterus. However, the expression and significance of MTSS1 have not been reported in precancerous cervical lesions or cervical cancer. In the present study, MTSS1 protein expression in CIN, cervical cancer and normal cervical tissues was examined by immunohistochemistry. The results revealed negative or weak expression of MTSS1 in the majority of normal cervical specimens. The MTTS1 positive expression rate and MTSS1 intensity were gradually increased along with an increased degree of precancerous cervical lesions. The positive expression rate of MTSS1 was significantly higher in CIN II–III and cervical cancer tissues than in CIN I and normal cervical tissues (P<0.05), suggesting that MTSS1 may be important in cervical carcinogenesis. Furthermore, the strong positive expression of MTSS1 was significantly higher in middle- and advanced-stage cervical cancer than in early stage cancer (P<0.05). This indicates that MTSS1 may be involved in cervical cancer invasion and metastasis, a finding consistent with those from studies regarding hepatocellular carcinoma and colorectal cancer tissues (13,14). Studies examining hepatocellular carcinoma and colon cancer also revealed that the expression of MTSS1 was positively associated with lymph node metastasis (9,10), but the results from the present study demonstrate no statistically significant difference in the MTSS1 expression levels between patients with lymphatic metastasis and those without. The lack of significance may be due to the limited number of cervical cancer samples; therefore, further confirmation in a larger sample group is required.

The mechanism by which MTSS1 is involved in cervical cancer development remains unclear. However, certain previous studies have provided insight into the possible underlying mechanisms. Wang *et al* (17) observed that tyrosine phosphorylation of the MTSS1 protein may be involved in platelet-derived growth factor-mediated cell shape changes. Therefore, MTTS1 may be part of a novel signaling pathway that connects platelet-derived growth factor signaling and the actin cytoskeleton through the tyrosine kinase, Src, which regulates novel actin-related proteins, such as MTSS1 (18). Another possible mechanism involves the potential association of MTSS1 expression in cervical cancer with hedgehog (Hh)-Gli signaling pathways. A previous study demonstrated that MTSS1 is a novel Hh-Gli signal response gene implicated in Gli regulation of tumorigenesis (19). Furthermore, Xuan *et al* (20) demonstrated active Hh-Gli signaling in cervical cancer and that expression levels of the Hh-Gli signaling protein were higher in CIN than in normal cervical tissue, suggesting that MTSS1 may be involved in the Hh-Gli signaling pathway through interaction with Gli, allowing the occurrence and development of cervical lesions. In addition, in head and neck squamous cell carcinoma cells, MTSS1 was found to interact with the epidermal growth factor (EGF), strengthen the localization of the EGF receptor in the cell membrane, prolong the Erk signal and promote cell proliferation (15). In cervical cancer, EGF was found to promote lamellipodial stretching by autocrine or paracrine mechanisms, thus promoting cervical cancer cell invasion (21). Elucidation of the specific mechanism of MTSS1 involvement in cervical cancer requires further studies using cervical cancer cell lines.

In conclusion, in the present study, MTSS1 was found to be highly expressed in CIN II–III and cervical cancer tissues, and MTSS1 expression levels were positively correlated with the clinical stage of cervical cancer. This provides a basis for further investigation of the biological role of MTSS1 in the development and progression of cervical cancer. MTSS1 may be a novel diagnostic biomarker or a therapeutic target in cervical cancer.

## Figures and Tables

**Table I:** Cytoplasmic MTSS1 expression in cervical tissues from each group.

		MTSS1 expression				
		
Group	n	−, n	+, n	++, n	+++, n	Positive rate, %
Normal cervix	30	24	6	0	0	20.00^a^
CIN I	30	17	11	2	0	43.33^b^
CIN II–III	30	0	2	15	13	100.00^c^
Cervical cancer	57	0	0	20	37	100.00^d^

a P>0.05 vs. CIN I (χ^2^=3.774, P=0.052);

b P<0.01 vs. CIN II–III (χ^2^=23.721, P<0.001);

c P<0.01 vs. normal cervix (χ^2^=40.000, P<0.001);

d P<0.01 vs*.* normal cervix (χ^2^=62.971, P<0.001).

MTSS, metastasis suppressor 1; CIN, cervical intraepithelial neoplasia.

**Table II:** Associations between MTSS1 expression and clinicopathological factors in cervical cancer patients.

		MTSS1 expression				
		
Clinicopathological factor	n	−, n	+, n	++, n	+++, n	Strong positive expression, %
Age, years						
<60	35	0	0	13	22	62.86^a^
≥60	22	0	0	7	15	68.18
Clinical stage						
I–IIa	23	0	0	14	9	39.13^b^
IIb–IV	34	0	0	6	28	82.35
Differentiation						
Well	12	0	0	5	7	58.33^c^
Moderate to low	45	0	0	15	30	66.67
Lymphatic metastasis						
Negative	48	0	0	17	31	64.58^d^
Positive	9	0	0	3	6	66.67

a P>0.05 (χ^2^=0.168, P=0.682);

b P<0.05 (χ^2^=11.253, P=0.01);

c P>0.05 (χ^2^=0.289, P=0.591);

d P>0.05 (χ^2^=0.014, P=0.904).

MTSS, metastasis suppressor 1.
